# Preconditioning beef cattle for long-duration transportation stress with rumen-protected methionine supplementation: A nutrigenetics study

**DOI:** 10.1371/journal.pone.0235481

**Published:** 2020-07-02

**Authors:** Gastón F. Alfaro, Taylor E. Novak, Soren P. Rodning, Sonia J. Moisá

**Affiliations:** Department of Animal Sciences, Auburn University, Auburn, AL, United States of America; University of Illinois, UNITED STATES

## Abstract

In general, beef cattle long-distance transportation from cow-calf operations to feedlots or from feedlots to abattoirs is a common situation in the beef industry. The aim of this study was to determine the effect of rumen-protected methionine (RPM) supplementation on a proposed gene network for muscle fatigue, creatine synthesis (*CKM*), and reactive oxygen species (ROS) metabolism after a transportation simulation in a test track. Angus × Simmental heifers (n = 18) were stratified by body weight (408 ± 64 kg; BW) and randomly assigned to dietary treatments: 1) control diet (CTRL) or 2) control diet + 8 gr/hd/day of top-dressed rumen-protected methionine (RPM). After an adaptation period to Calan gates, animals received the mentioned dietary treatment consisting of Bermuda hay *ad libitum* and a soy hulls and corn gluten feed based supplement. After 45 days of supplementation, animals were loaded onto a trailer and transported for 22 hours (long-term transportation). *Longissimus* muscle biopsies, BW and blood samples were obtained on day 0 (Baseline), 43 (Pre-transport; PRET), and 46 (Post-transport; POST). Heifers’ average daily gain did not differ between baseline and PRET. Control heifer’s shrink was 10% of BW while RPM heifers shrink was 8%. Serum cortisol decreased, and glucose and creatine kinase levels increased after transportation, but no differences were observed between treatments. Messenger RNA was extracted from skeletal muscle tissue and gene expression analysis was performed by RT-qPCR. Results showed that *AHCY* and *DNMT3A* (DNA methylation), *SSPN* (Sarcoglycan complex), and *SOD2* (Oxidative Stress-ROS) were upregulated in CTRL between baseline and PRET and, decreased between pre and POST while they remained constant for RPM. Furthermore, *CKM* was not affected by treatments. In conclusion, RPM supplementation may affect ROS production and enhance DNA hypermethylation, after a long-term transportation.

## Introduction

Long-distance road transportation (> 400 km) is a stressful event that most cattle experience at least once in their lifetime [1]. It is important to consider that road transportation includes more than just the shipping itself; it should also considerthe assembly and loading of animals at their place of origin, confinement on a moving or stationary vehicle, unloading, and penning at their final destination [2]. Several research studies have been conducted in the last few decades, in an effort to achieve a better understanding of the characteristics of road transportation and its effects on cattle [3–6]. Analysis of these effects will improve animal welfare and help reduce the economic losses associated with transportation [7].

Cattle have to potentially stand for long periods of time during transportation, thus muscles are constantly in tension [8]. Cattle transportation in the United States usually takes many hours, since the distance between cow-calf operations or stocker operations and feedlots is considerable [9]. One of the negative effects of long-term transportation is muscle fatigue, which can be defined as the reversible decrease of force generation by a muscle after its intense and repetitive use. In other words, muscle fatigue manifests as an inability to continue a motor task at the required intensity, eventually leading to exhaustion [10].

**Fig 1:**
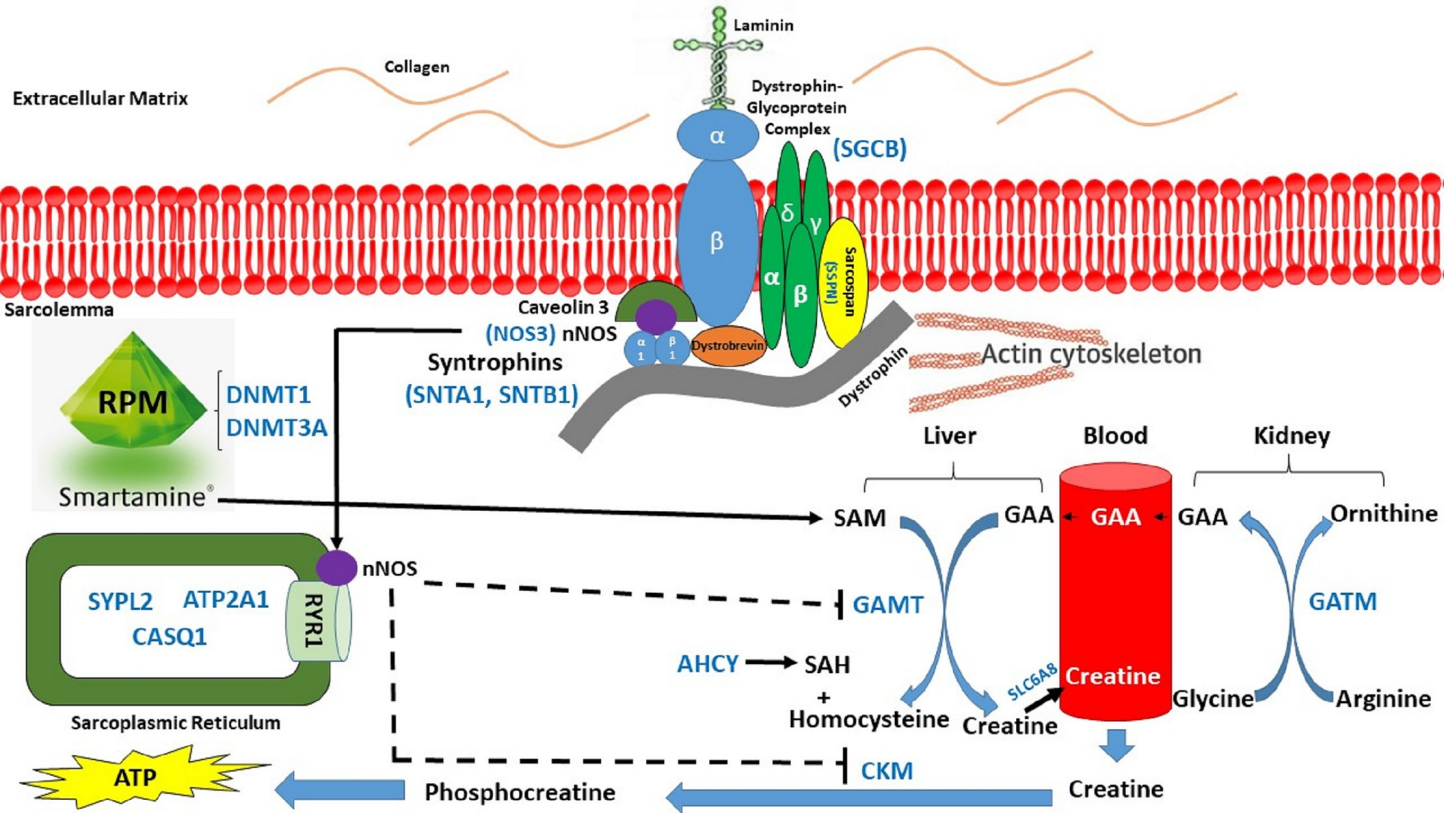
The proposed effect of administration of RPM on alleviation of muscle fatigue in beef cattle stressed due to long-duration transportation.

Our hypothesis was that rumen-protected methionine (RPM) supplementation during a preconditioning period of 45 days prior to transportation could provide additional methyl groups required for the activation of the creatine precursor called S-Adenosyl methionine (SAM), which will generate additional energy (ATP) required to mitigate muscle fatigue [11]. The proposed muscle fatigue gene network is as follows (Fig 1): RPM provided by Smartamine® will incorporate additional methyl groups required for the synthesis of creatine in the liver by guanidinoacetate N-methyltransferase (*GAMT*). Glycine amidinotransferase, mitochondrial (*GATM*) is involved in creatine biosynthesis, whereby it catalyzes the transfer of a guanido group from L-arginine to glycine, resulting in guanidinoacetic acid (*GAA*), the immediate precursor of creatine. In skeletal muscle, the dystroglycan complex works as a transmembrane linkage between the extracellular matrix and the cytoskeleton. α-dystroglycan is extracellular and binds to laminin in the basement membrane, while β-dystroglycan is a transmembrane protein and binds to dystrophin. Dystrophin attaches to intracellular actin cables. In this way, the dystroglycan complex, which links the extracellular matrix to the intracellular actin cables, is thought to provide structural integrity in muscle tissues. The neuronal form of nitric oxide (*nNOS*) inhibits *GAMT* and the enzyme creatine kinase present in muscle (*CKM*), responsible for the conversion of creatine to phosphocreatine which releases the energy (ATP) required to cope against muscle fatigue (Fig 1).

The objective of this study was to analyze the effect of administration of RPM on a proposed muscle fatigue gene network during pre- and post- exposure to a long-duration transportation simulation. Furthermore, we aimed to determine if RPM induces changes in expression of genes related to oxidative stress and DNA methylation.

## Materials and methods

All the procedures for this study were conducted in accordance with a protocol approved by the Institutional Animal Care and Use Committee of Auburn University (IACUC Protocol #2017‐3129). Eighteen Angus × Simmental heifers were divided in two groups of 9 each, with the same average body weight (BW; 408 ± 64 kg) and age (375 ± 44 days old) per treatment. Animals belonged to the E. V. Smith Research Center Beef Unit located in Shorter, AL (36075), and they were relocated to the Beef Cattle Evaluation Center, Auburn University, Auburn, AL (36849) for the duration of the study. After a successful 21-day adaptation period to a Calan Gate System (Northwood, NH), both groups received two pounds per day of a diet consisting of soyhulls (50%) and corn gluten feed (50%) with bermudagrass hay *ad libitum*. Additionally, during the following 45 days, nine heifers received supplement containing 8 gr/hd/day as a fixed rate of top-dressed RPM (Smartamine M, Adisseo Inc., Antony, France), whereas the remaining nine heifers received supplement without the addition of RPM (CTRL).

After 45 days on treatment, both groups were loaded onto a 32’ × 7’ steel gooseneck trailer (Circle W trailers, McKenzie, AL 36456) at a stocking density of 1.15 m^2^/hd. Subsequently, animals were transported via pickup truck and trailer from the Beef Cattle Evaluation Center located at 405 Shug Jordan Parkway, Auburn, AL 36832 to the National Center for Asphalt Technology (NCAT) Test Track located at 1600 Lee Road 151, Opelika, AL 36804 (~18 miles apart). The transportation simulation took place on a 1.7-mile oval test track at NCAT, where animals remained loaded and transported for 22 hours, at a constant speed of 45 mph. A team of three truck drivers followed a modified version of the NCAT driver safety schedule which consisted of stops once per hour (5 minutes per stop) to rest, and every three hours for fuel (~10 minutes per fuel stop). Every time the truck stopped, visual evaluations of the animals were performed to check their well-being (i.e. panting, respiration rate, proportion of heifers resting or standing).

Animals were weighed at the beginning of the adaptation period to Calan Gate System (Day– 21), at the beginning of the study (Day 0), 2 days before transportation (Day 43, pre-transportation, PRET), and immediately after transportation (Day 46, post-transportation, POST).

The first skeletal muscle biopsy was performed at the beginning of the administration of RPM (Day 0 or baseline—BASE). The second biopsy was performed 2 days prior to transportation simulation (PRET), and a final biopsy was performed right after unloading the trailer following transportation simulation (POST). These biopsies were taken in order to assess muscle fatigue-related gene expression changes due to RPM supplementation throughout the preconditioning period, during and after transportation. Animals’ IDs were recorded so that the same animals were biopsied at each time point to provide repeated measures sampling.

**Fig 2:**
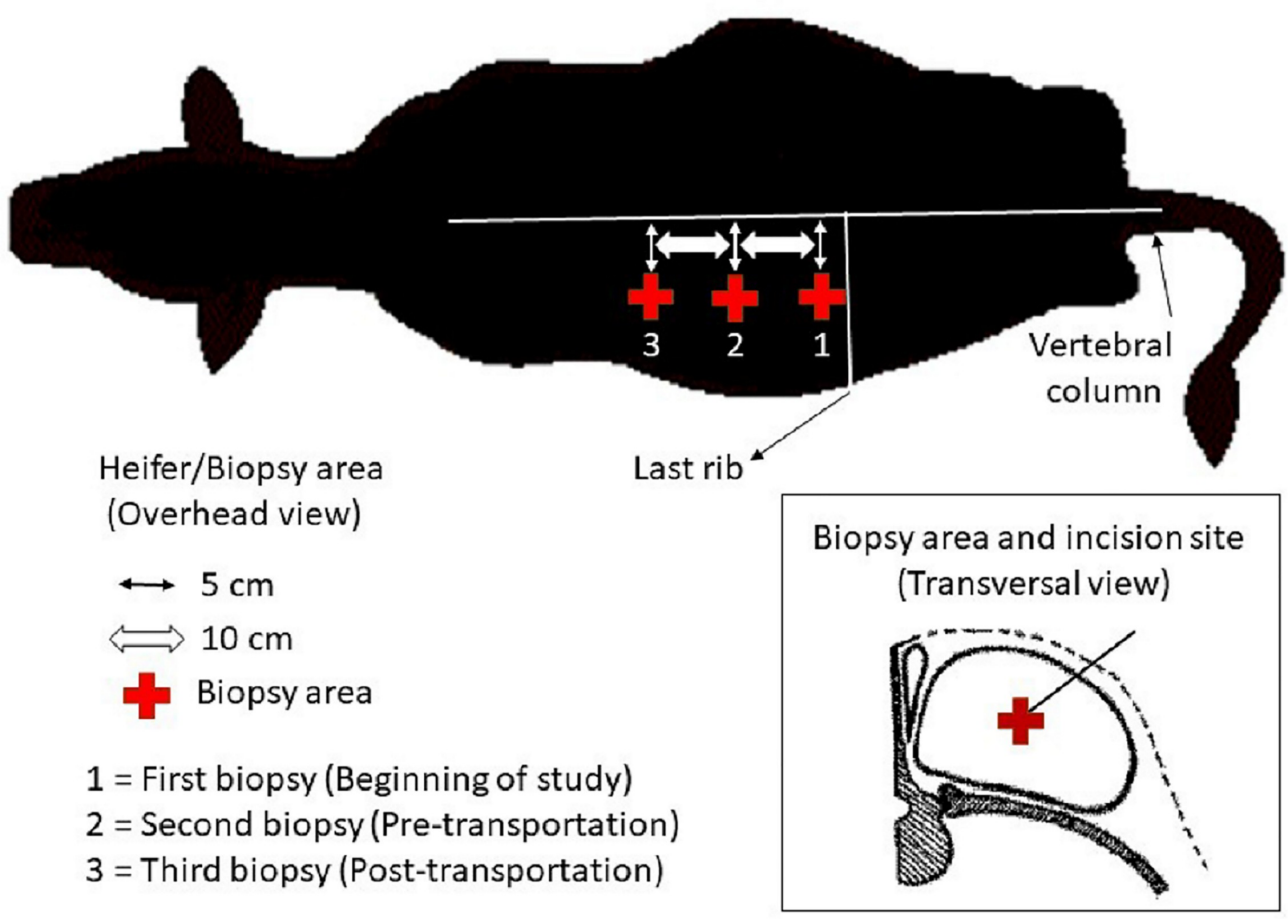
Overhead and transversal view of skeletal muscle biopsy area on a beef heifer.

Skeletal muscle biopsies of *Longissimus dorsi* muscle were performed for epigenetic regulation (DNA methylation) and gene expression analysis using real-time quantitative PCR (RT-qPCR). Each muscle sample was obtained from the left side and moving up the side toward the head (longitudinally) with each sequential biopsy. Anesthesia injections of 5 mL of Lidocaine HCl 2% (VetOne®, Boise, ID) were placed before perform each biopsy incision. A muscle biopsy core (500–600 mg) was removed using a biopsy needle (12-gauge × 16 cm) inserted in a biopsy gun (Bard Magnum MG#1522, Tempe, AZ). The first biopsy area coincided with the *Longissimus dorsi* muscle at the level of the last rib. The second biopsy was performed 10 cm towards the head from the first biopsy area, and the final biopsy was 10 cm towards the head from the second biopsy area. The incision sites for all biopsies were located 5 cm down the vertebral column with the aim to take samples from the center of the *Longissimus dorsi* muscle (Fig 2). Ten mL blood samples were collected via jugular venipuncture at BASE, PRET and POST to determine hormonal and metabolic changes. Cortisol concentrations were measured at the Auburn University Endocrine Diagnostics Laboratory with the IMMULITE 2000 (Siemens Healthcare Diagnostics, Deerfield, IL, USA), which uses a solid-phase competitive enzyme-amplified chemiluminescent immunoassay. Creatine kinase and glucose analysis were performed at Auburn University Clinical Pathology Laboratory using methods previously described [12, 13].

The protocols used for RNA extraction, primer design and testing, cDNA synthesis and RT-qPCR analysis were previously described [14]. RNA integrity number average was 7 ± 0.5. The following genes were selected (S1 Table): as internal controls: Mitochondrial Ribosome-Associated GTPase 1 (*MTG1)*, Ribosomal Protein S15a (*RPS15A*) and, Ubiquitously Expressed Prefoldin Like Chaperone (*UXT*). Furthermore, for the dystrophin-glycoprotein complex, we selected Sarcoglycan Beta (*SGCB*), Syntrophin alpha 1 (*SNTA1*), Syntrophin beta 1 (*SNTB1*) and Sarcospan (*SSPN*). For genes present in the sarcoplasmic reticulum, we selected ATPase Sarcoplasmic/Endoplasmic Reticulum Ca^++^ Transporting 1 (*ATP2A1*), Calsequestrin (*CASQ1*) and Synaptophysin Like 2/MG29 (*SYPL2*). For the creatine synthesis pathway, we considered Creatine Kinase M-type (*CKM*), Guanidinoacetate N-methyltransferase (*GAMT*), Glycine amidinotransferase (*GATM*) and, Solute carrier family 6 member 8 (*SLC6A8*). For genes related to oxidative stress, we selected the following genes: Superoxide dismutase 1 (*SOD1*), Superoxide dismutase 2 (*SOD2*), Nitric Oxide synthase 3 (*NOS3*), Nuclear factor kappa B subunit 1 (*NFKB1*), NAD(P)H quinone dehydrogenase 1 (*NQO1*) and, PPARg coactivator alpha (*PGC1a*). Finally, for DNA methylation we selected DNA Methyltransferase 1 (*DNMT1*), DNA Methyltransferase 3 alpha (*DNMT3A*) and Adenosylhomocysteinase (*AHCY*). Further information is available in supporting tables (S2–S5 Tables).

### Statistical analysis

Real time quantitative PCR data were analyzed using the MIXED procedure of SAS (SAS 9.4 Institute, Cary, NC, USA). Before statistical analysis, RT-qPCR data was normalized using quantities values of expression of each gene under study, divided by the geometric mean of 3 housekeeping genes (i.e., *UXT*, *MTG1* and *RPS15A*). Then, normalized data was transformed to fold-change relative to day 0 (i.e. BASE). To estimate standard errors at day 0 and prevent biases in statistical analysis, normalized RT-qPCR data were transformed to obtain a perfect mean of 1.0 at day 0, leaving the proportional difference between the biological replicate. The same proportional change was calculated at all other time points to obtain a fold-change relative to day 0. Fixed effects in the statistical model for each variable analyzed (i.e. genes, blood metabolite) included treatment, time on experiment and treatment × time on experiment interactions when appropriate (e.g. mRNA expression over time). Gene expression data analysis included a repeated-measures statement with an autoregressive covariate structure. Animal performance and blood metabolite levels were also analyzed using the MIXED procedure of SAS, and treatment was the fixed effect in the statistical model. The random effect in all models was heifer within treatment. Average daily gain data was analyzed using PROC GLM procedure of SAS.

The statistical model used was: *Yijl* = *μ + Ci + Tj + Sl + (C × T)ij + εijl*; where, Y_ijl_ is the background-adjusted normalized fold change or blood data value; μ is the overall mean; C_i_ is the fixed effect of time (3 levels); T_j_ is the fixed effect of treatment (2 levels); S_l_ is the random effect of heifer nested within treatment; C × T is the interactions of time by treatment and ε_ijl_ is the random error (0, σ_e_^2^) associated with Y_ijl_.

## Results

### Animal performance

**Fig 3:**
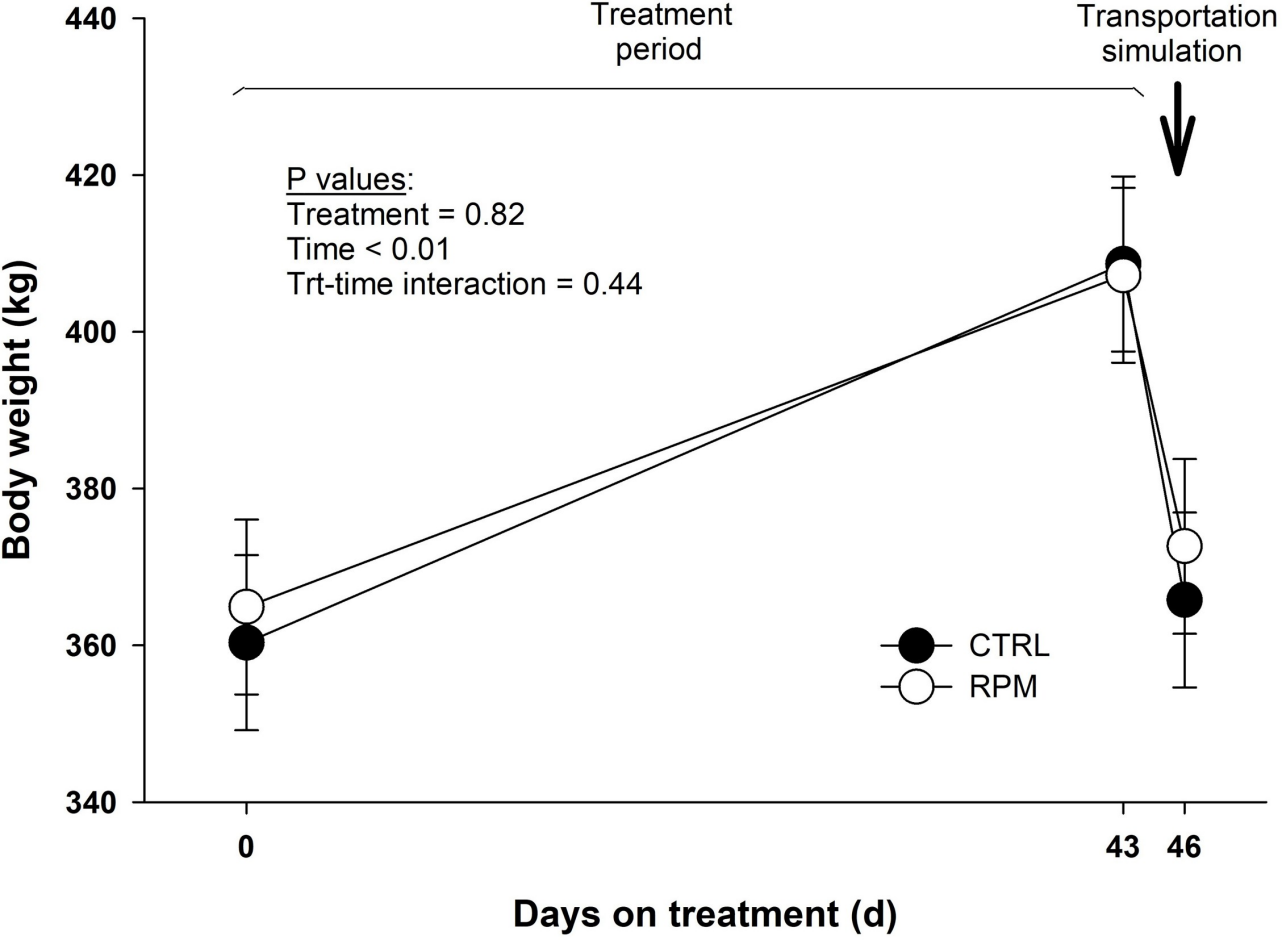
Body weight of beef heifers that received rumen-protected methionine (RPM) and heifers control (CTRL) that did not received RPM. Day 0 (BASE), day 43 (PRET) and day 46 (POST). Statistical significant differences where declared at *P* < 0.05 and tendencies at *P* > 0.05 and < 0.1.

Body weight did not present a treatment × time interaction (*P* = 0.44), although it showed a decrease after transportation in CTRL and RPM heifers (*P* < 0.01) (Fig 3). Shrink loss was calculated by difference in body weight before and after transportation. Control heifers lost 43 kg (10% shrink), while RPM heifers lost 35 kg (8% shrink). The *P* values for shrink loss in kilograms and by percent were not significant (*P* = 0.29 and *P* = 0.28, respectively). Furthermore, average daily gain (ADG) did not differ between treatments (*P* = 0.41) during the preconditioning period (i.e., between BASE and PRET). Heifers in RPM group had an ADG of 0.98 kg/day, whereas heifers in CTRL an ADG of 1.12 kg/day(S1 Fig).

### Serum cortisol, glucose, and creatine kinase

**Fig 4:**
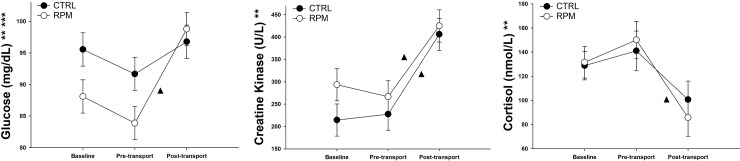
Serum glucose, creatine kinase and cortisol levels of beef heifers that received rumen-protected methionine (RPM) for 45 days before transportation and heifers control (CTRL) that did not received RPM. Statistically significant differences were declared at *P* < 0.05 and tendencies at *P* > 0.05 and < 0.1.

Glucose levels showed a treatment × time interaction (*P* < 0.05) and a time effect (*P* < 0.05). There was not a significant difference (*P* = 0.14) in glucose concentration in both treatments between BASE and PRET Heifers in RPM group had a significant increase (*P* < 0.05) in glucose levels between PRET (83.9 mg/dL) and POST (98.8 mg/dL); and heifers in CTRL did not show a significant difference between PRET (91.7 mg/dL), and POST (96.8 mg/dL) (Fig 4).

Circulating creatine kinase levels did not show a treatment × time interaction (*P* = 0.16). However, creatine kinase concentration showed a time effect (*P* < 0.05). Both groups showed a significant increase between PRET and POST (Fig 4). Finally, serum cortisol concentrations did not present a treatment × time interaction (*P* = 0.68). However, there was an important reduction in cortisol levels after transportation in CTRL and RPM heifers (Fig 4).

### Gene expression

#### Dystrophin-glycoprotein complex

**Fig 5:**
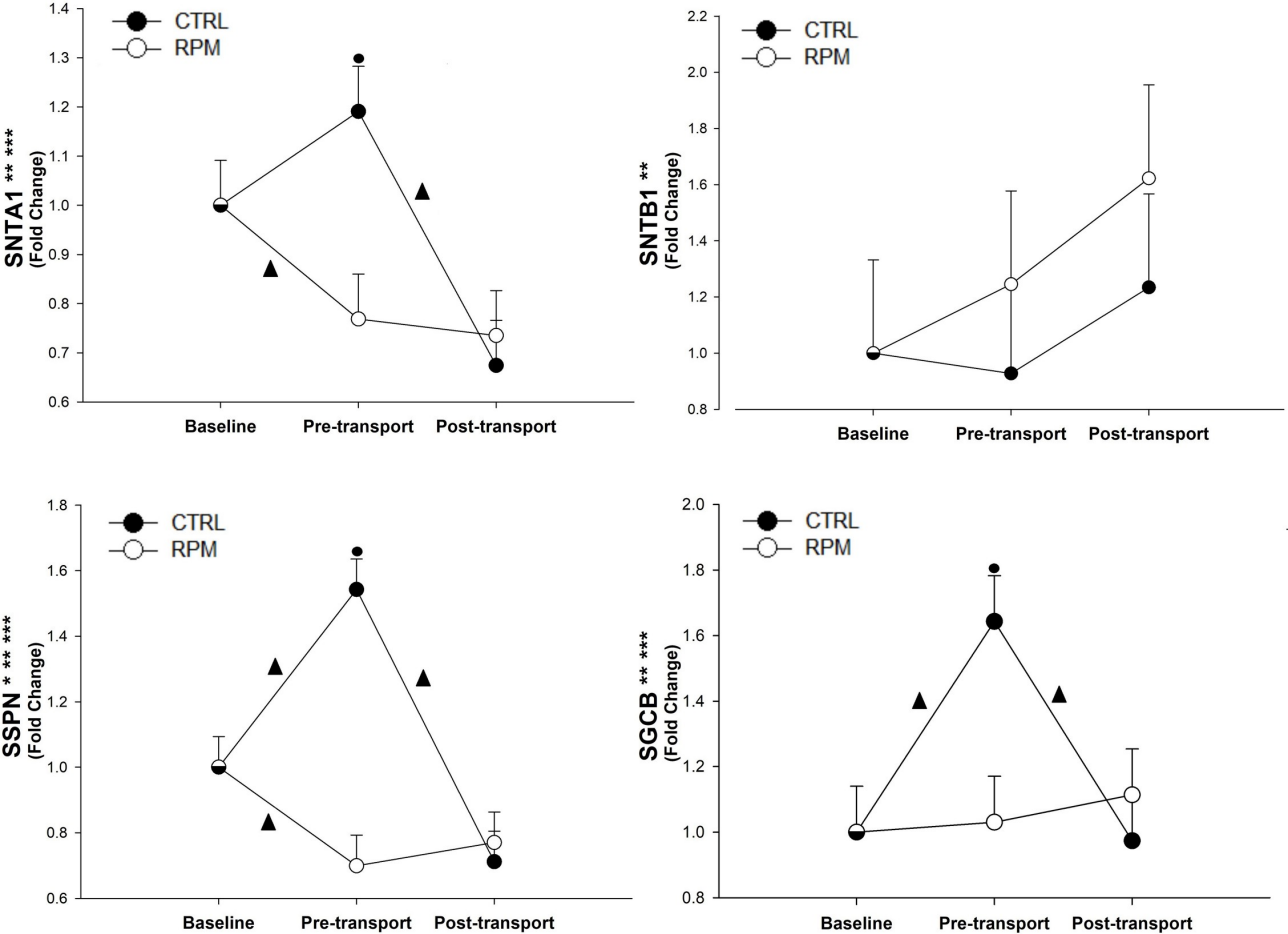
Expression of genes related to the dystrophin-glycoprotein complex in *Longissimus dorsi* muscle of beef heifers that received rumen-protected methionine (RPM) for 45 days before transportation and heifers control (CTRL) that did not received RPM. Statistically significant differences were declared at *P* < 0.05 and tendencies at *P* > 0.05 and < 0.1. Treatment × time interaction (***), time effect (**) and treatment effect (*). Tendencies are denoted if symbols (*, ** or ***) are underlined. Symbols (▲) on lines denote significant differences (*P* < 0.05) between two time points for the same treatment, symbols (●) denote significant differences (*P* < 0.05) between treatment at the same time point. Because the data presented in this chart are fold-change values, the baseline reference value is 1 for RPM and CTRL, therefore, there is overlap between symbols with error bars at baseline.

There was a treatment × time interaction (*P* < 0.05) and time effect (*P* < 0.05) for *SGCB*, *SNTA1* and *SSPN* (Fig 5). The mRNA expression of *SGCB* in CTRL group was upregulated between BASE and PRET (*P* < 0.05), and then it was decreased between PRET and POST (*P* < 0.05). In RPM treatment, *SNTA1* mRNA expression was downregulated between BASE and PRET (*P* < 0.05) and downregulated in CTRL between PRET and POST (*P* < 0.05). Expression of *SSPN* had a treatment × time interaction (*P* < 0.01), treatment effect (*P* < 0.05), and time effect (*P* < 0.01). Its expression was upregulated in CTRL between BASE and PRET (*P* < 0.05), and then downregulated between PRET and POST (*P* < 0.05). However, *SSPN* expression decreased in RPM between BASE and PRET (*P* < 0.05). The mRNA expression of *SGCB*, *SSPN*, and *STNA1* in CTRL were greater than RPM (*P* < 0.05) at PRET. Finally, *SNTB1* did not showed a significant treatment × time interaction (*P* = 0.34) (Fig 5).

#### Sarcoplasmic reticulum

**Fig 6:**
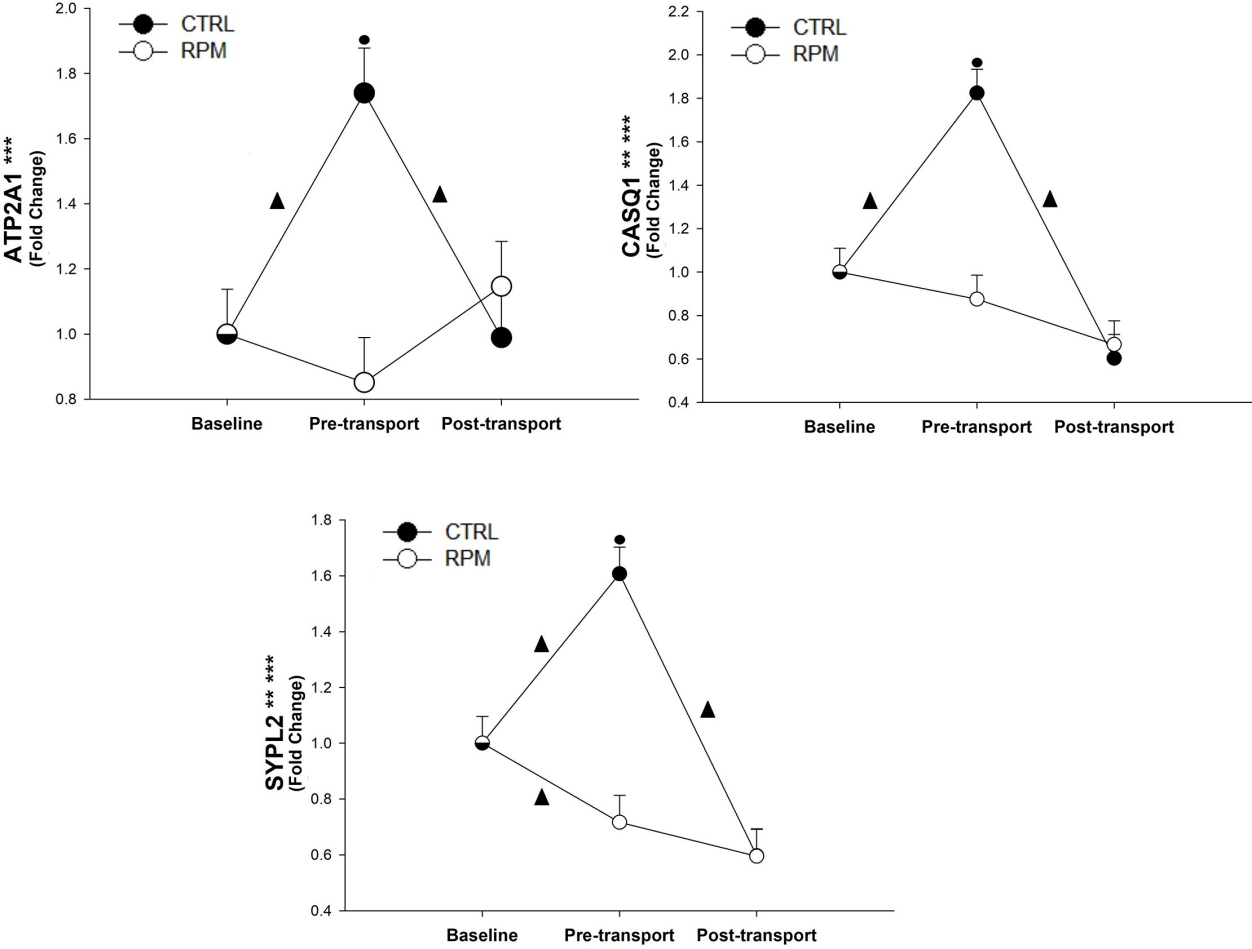
Expression of genes located at the sarcoplasmic reticulum in *Longissimus dorsi* muscle of beef heifers that received rumen-protected methionine (RPM) for 45 days before transportation and heifers control (CTRL) that did not received RPM. Statistically significant differences were declared at *P* < 0.05 and tendencies at *P* > 0.05 and < 0.1. Treatment × time interaction (***), time effect (**) and treatment effect (*). Tendencies are denoted if symbols (*, ** or ***) are underlined. Symbols (▲) on lines denote significant differences (*P* < 0.05) between two time points for the same treatment, symbols (●) denote significant differences (*P* < 0.05) between treatment at the same time point. Because the data presented in this chart are fold-change values, the baseline reference value is 1 for RPM and CTRL, therefore, there is overlap between symbols with error bars at baseline.

There was a treatment × time interaction (*P* < 0.01) and a time effect (*P* < 0.01) for both *CASQ1* and *SYPL2* (Fig 6). Furthermore, *ATP2A1* showed only a treatment × time interaction (*P* < 0.01). In all cases, *ATP2A1*, *CASQ1* and *SYPL2* mRNA expression for CTRL was significantly greater (*P* < 0.05) than RPM at PRET. Furthermore, *ATP2A1*, *CASQ1* and *SYPL2* expressions for CTRL increased (*P* < 0.05) between BASE and PRET; however, their level of expression decreased (*P* < 0.05) between PRET and POST. In addition, *SYPL2* had a significant downregulation (*P* < 0.05) between BASE and PRET in RPM heifers (Fig 6).

#### Creatine synthesis

**Fig 7:**
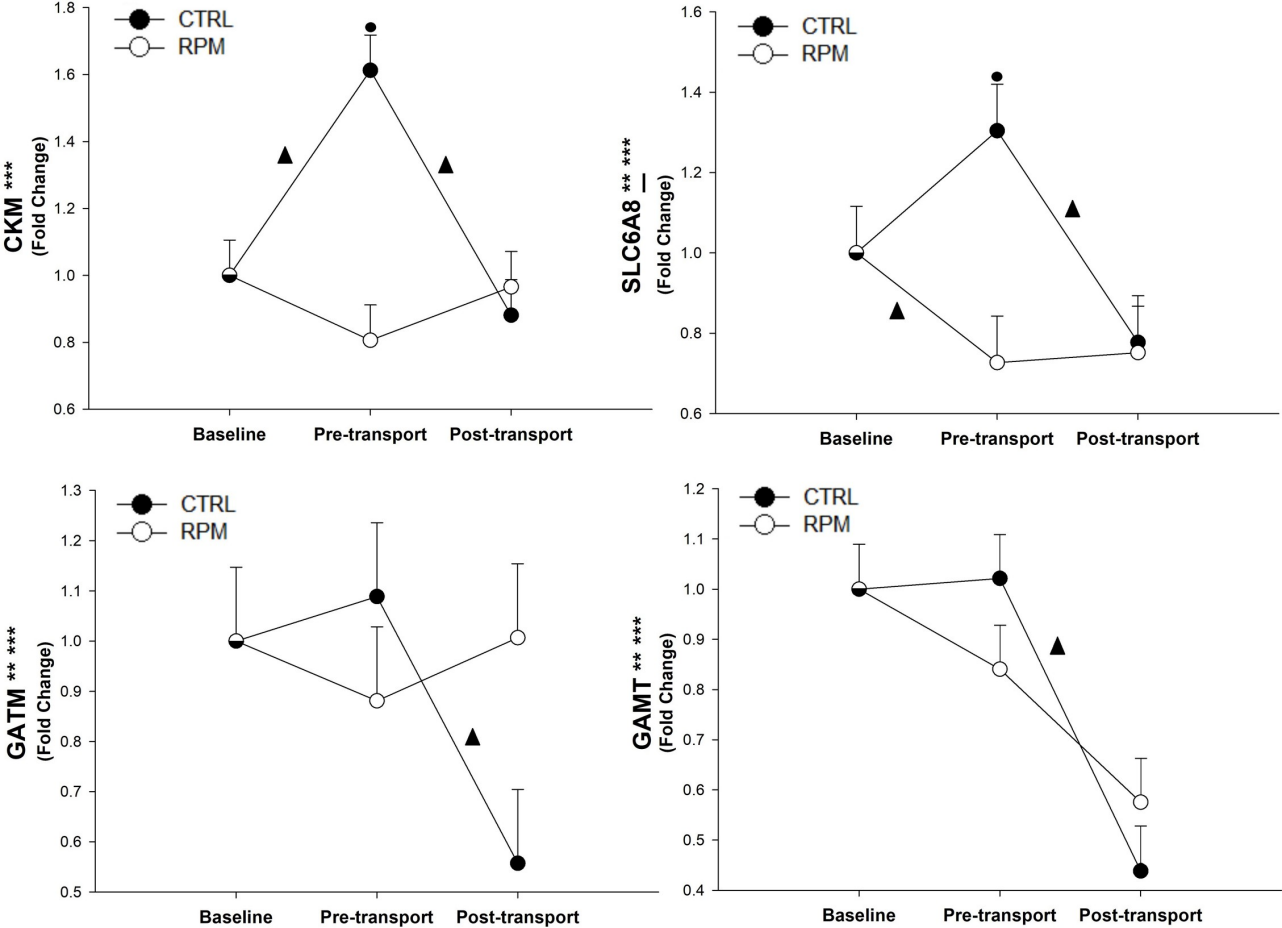
Expression of genes related to the creatine synthesis pathway in *Longissimus dorsi* muscle of beef heifers that received rumen-protected methionine (RPM) for 45 days before transportation and heifers control (CTRL) that did not received RPM. Statistically significant differences were declared at *P* < 0.05 and tendencies at *P* > 0.05 and < 0.1. Treatment × time interaction (***), time effect (**) and treatment effect (*). Tendencies are denoted if symbols (*, ** or ***) are underlined. Symbols (▲) on lines denote significant differences (*P* < 0.05) between two time points for the same treatment, symbols (●) denote significant differences (*P* < 0.05) between treatment at the same time point. Because the data presented in this chart are fold-change values, the baseline reference value is 1 for RPM and CTRL, therefore, there is overlap between symbols with error bars at baseline.

Expression of *CKM* showed a significant treatment × time interaction (*P* < 0.01). The mRNA expression of *GAMT* and *GATM* showed a treatment × time interaction (*P* < 0.05) and a time effect (*P* < 0.01). The mRNA expression of *SLC6A8* showed a treatment × time interaction (*P* < 0.01) and a tendency for a time effect (*P* < 0.08). In addition, *CKM* and *SLC6A8* mRNA expression on CTRL was greater (*P* < 0.05) than RPM at PRET. Finally, CKM expression was increased (*P* < 0.05) between BASE and PRET in CTRL, but its expression was decreased (*P* < 0.05) between PRET and POST. In CTRL animals, *GATM* and *GAMT* expressions were downregulated (*P* < 0.05) between PRET and POST. In RPM heifers, *SLC6A8* expression was decreased (*P* < 0.05) and increased in CTRL heifers (*P* < 0.05) between BASE and PRET, while *SLC6A8* decreased between PRET and POST for CTRL but did not change for RPM. Finally, *SLC6A8* had a tendency for a time effect (*P* = 0.07) (Fig 7).

#### DNA methylation

There was a treatment × time interaction (*P* = 0.05), and a time effect (*P* < 0.01) for *DNMT1* mRNA expression. Furthermore, *DNMT3A* showed a treatment × time interaction (*P* < 0.01), a treatment effect (*P* = 0.05), and a time effect (*P* < 0.01). Furthermore, there was an increase of *DNMT1* mRNA expression in RPM between PRET and POST. For *DNMT3A*, CTRL had a greater expression (*P* < 0.05) at PRET than RPM. Finally, *DNMT3A* mRNA expression decreased (*P* < 0.05) in RPM between BASE and PRET. Similarly, *DNMT3A* expression was downregulated (*P* < 0.05) in CTRL between PRET and POST.

**Fig 8:**
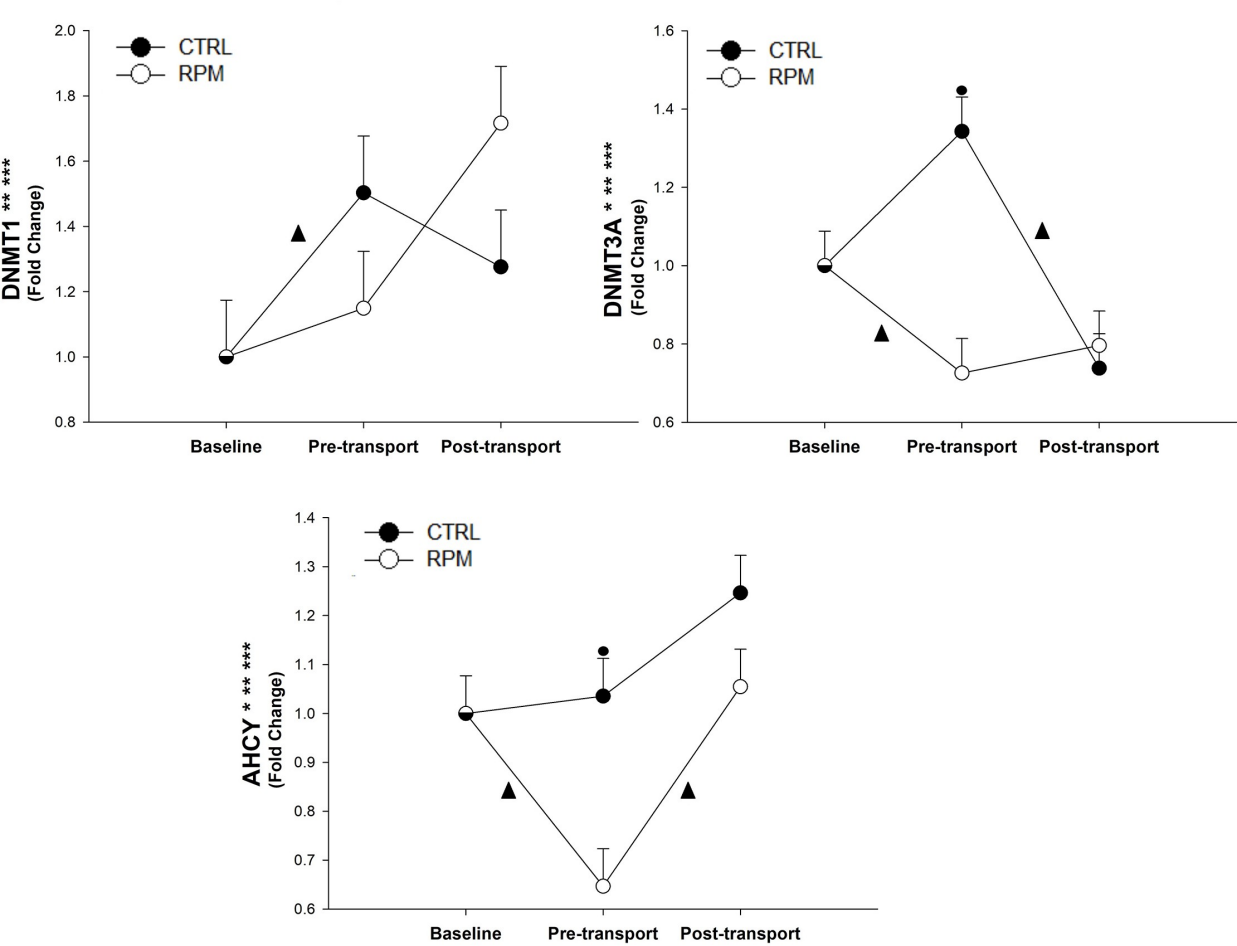
Expression of genes related to DNA methylation in *Longissimus dorsi* muscle of beef heifers that received rumen-protected methionine (RPM) for 45 days before transportation and heifers control (CTRL) that did not received RPM. Statistically significant differences were declared at *P* < 0.05 and tendencies at *P* > 0.05 and < 0.1. Treatment × time interaction (***), time effect (**) and treatment effect (*). Tendencies are denoted if symbols (*, ** or ***) are underlined. Symbols (▲) on lines denote significant differences (*P* < 0.05) between two time points for the same treatment, symbols (●) denote significant differences (*P* < 0.05) between treatment at the same time point. Because the data presented in this chart are fold-change values, the baseline reference value is 1 for RPM and CTRL, therefore, there is overlap between symbols with error bars at baseline.

There was a significant treatment × time interaction (*P* < 0.01), treatment effect (*P* < 0.05), and time effect (*P* < 0.01) for *AHCY* mRNA expression. In addition, *AHCY* mRNA expression in CTRL was greater (*P* < 0.05) than RPM at PRET. There was a significant downregulation of *AHCY* (*P* < 0.05) in RPM between BASE and PRET; however, it was significantly upregulated (*P* < 0.05) between PRET and POST (Fig 8).

#### Oxidative stress

**Fig 9:**
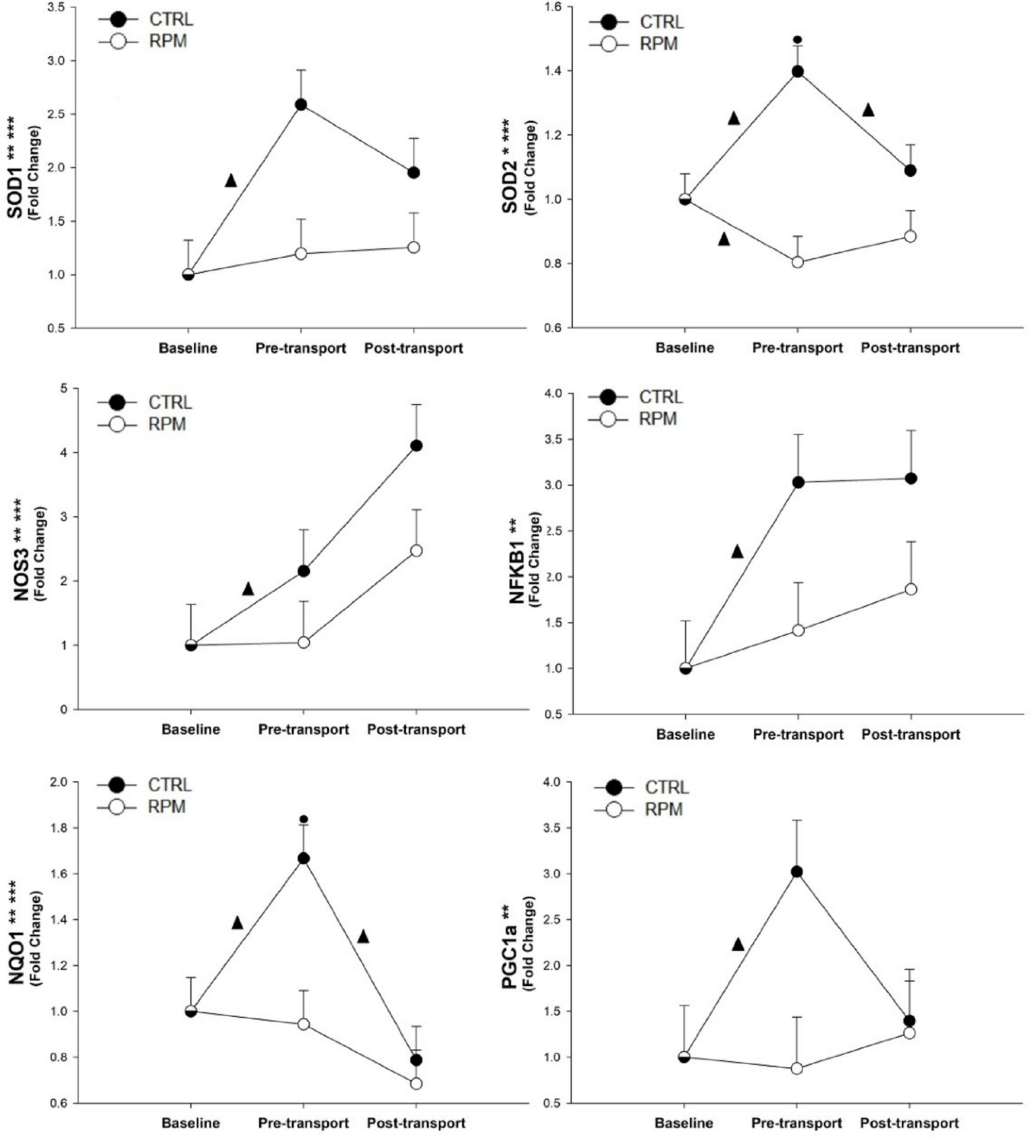
Expression of genes related to oxidative stress in *Longissimus dorsi* muscle of beef heifers that received rumen-protected methionine (RPM) for 45 days before transportation and heifers control (CTRL) that did not received RPM. Statistically significant differences were declared at *P* < 0.05 and tendencies at *P* > 0.05 and < 0.1. Treatment × time interaction (***), time effect (**) and treatment effect (*). Tendencies are denoted if symbols (*, ** or ***) are underlined. Symbols (▲) on lines denote significant differences (*P* < 0.05) between two time point for the same treatment, symbols (●) denote significant differences (*P* < 0.05) between treatment at the same time point. Because the data presented in this chart are fold-change values, the baseline reference value is 1 for RPM and CTRL, therefore, there is overlap between symbols with error bars at baseline.

There was a significant treatment × time interaction (*P* < 0.05), and a time effect (*P* < 0.01), for *SOD1*, *NQO1*, and *NOS3* mRNA expression. Furthermore, *SOD2* showed a significant treatment × time interaction (*P* < 0.01), and treatment effect (*P* < 0.05). There was not a significant treatment × time interaction (*P* > 0.05) for *PGC1α* and *NFKB1*. At PRET, CTRL had a greater expression (*P* < 0.05) for *SOD2*, and *NQO1* expressions in comparison to RPM. There was a significant increase (*P* < 0.05) in *SOD1*, *NOS3*, *NFKB1*, and *PGC1α* mRNA expressions in CTRL between BASE and PRET. In CTRL heifers, *SOD2* mRNA expression presented upregulation (*P* < 0.05) between BASE and PRET; however, its expression was downregulated between PRET and POST. In the case of *SOD2* expression in RPM, a significant decrease (*P* < 0.05) in expression was observed between BASE and PRET, which remained steady between PRET and POST. Finally, *NQO1* mRNA expression increased significantly (*P* < 0.05) in CTRL between BASE and PRET; however, a significant decrease (*P* < 0.05) was observed between PRET and POST, while it remained steady for RPM group (Fig 9).

## Discussion

### Animal performance

With respect to animal performance, CTRL group showed no difference in ADG compared with RPM group (1.12 kg/d vs. 0.98 kg/d, respectively). There are no previous studies that report the performance of beef heifers under RPM supplementation; however, our results support Chen et al. (2011), who evaluated RPM supplementation (Smartamine®) in dairy cows, and reported no difference in ADG with a control group [15]. In addition, Torrentera et al. (2017) tested the performance of Holstein steers (127 ± 4.9 kg) under the inclusion of 0.032%, 0.064%, 0.096%, or 0.128% of RPM. They obtained superior results with RPM supplementation when Smartamine® was included at a level of 0.096% of dietary dry matter (DM) [16]. In our study, the RPM supplementation was 0.07% of DM; inclusion up to 0.09% might possibly have results that are more notable.

The most important parameter to assess, from the beef production standpoint, is the body weight loss or shrink associated with transportation. Numerous factors affect shrink during livestock transportation. Transportation duration and distance travelled are especially critical. In our study, animals were loaded on the trailer for 22 hours; however, because of the stops to rest, the net transportation time was 20 hours. Interestingly, Lambooy & Hulsegge (1988) studied the effect of 24 hours transportation in pregnant heifers, and reported body weight losses of 8% on average [17]. This result is similar to the shrink loss we observed for RPM heifers.

Environmental conditions during the transportation simulation day was retrieved from AWIS (Agricultural Weather Information Service, Inc.) at the Auburn Mesonet weather station (Auburn_CR10_92–18, Lee County) which is located 5.2 miles from NCAT. The average temperature was 5°C, relative humidity was 48.5%, and no rain was recorded during the 22 hours of transportation nor on the previous or subsequent days. According to Gonzalez et al. (2012), 20–25 hours transportation with an ambient temperature of ~10°C will produce a ~8% of BW losses on feeder cattle, and ~10% of BW losses on cull cattle [18]. Thus, the environmental conditions during our trial were not detrimental for body weight losses. However, RPM supplementation may affect shrink during warmer seasons in the Southeast.

### Serum cortisol, glucose, and creatine kinase

Transportation is a stressful event that causes physiological and endocrine changes in animals’ metabolism. One indicator of these changes is cortisol, a glucocorticoid hormone released from the adrenal gland in response to stress [19]. Several studies have shown an increase in cortisol levels due to transportation in cattle, especially in the first hours of the trip [19, 20]. The release of blood cortisol increases when the transportation starts, reaching its highest point at ~ 4 hours, and then it starts to decline until reaching similar levels to the beginning of transportation. This level is maintained for the next 24 hours post-transportation [21]. However, these studies assessed the effect of shorter transportation time (i.e., 9 hours maximum), in comparison to a longer transportation period as in this study. Nevertheless, evidence of the reduction of blood cortisol level due to a long transportation could be associated to a familiarization of the animal with the environment inside the truck [22]. In total, our truck was stopped for 160 minutes during the 22-hour trip, either to rest or to refuel, and visual evaluations of the animals’ status took place during these stops. The small amount of time that the truck was stopped, could be beneficial for both, driver and animals. Frequent rest stops with loading and unloading may be more stressful than continuously being in the vehicle, consequently, this practice would likely be detrimental for the well-being of the animals [8].

Blood glucose level is used as a metabolic indicator of energy status in cattle. Our results suggest that glucose levels in blood during the preconditioning period are comparable to previous studies that reported 80.53 mg/dL of glucose in growing steers consuming a high forage diet [23]. Tarrant et al (1992) assessed the metabolic changes on Friesian steers after a 24-hour transportation. They reported PRET glucose levels of 89.2 mg/dL and POST glucose levels of 106.30 mg/dL, both of which are comparable to our results [24]. In addition, Earley et al (2010) reported blood glucose levels of growing Charolais bulls of 77.5 mg/dL at PRET, and 104.5 mg/dL immediately after a 24-hour transportation was complete [25]. However, the increase in glucose in RPM during transportation could be related to greater glycogenolysis, associated with the increase in catecholamine’s and glucocorticoids which were released after long-duration transportation stress [26].

Finally, creatine kinase (CK) in blood is commonly used in studies related to muscle fatigue for assessing the activity of the muscle [27, 28]. Even though, there are some differences in the serum CK levels in male and females [29], there is not a clear pattern. Previous studies reported increased CK activity due to transportation in cattle [24]. Tarrant et al (1992) reported 234 U/L of increase in CK after 24-hour transportation with a stocking density (1.13 m^2^/hd) similar to the one used in our study (1.15 m^2^/hd). However, we reported a lower increase in circulating CK during transportation for RPM and CTRL (*P* = 0.16), which may be related to the BW of the animals. Tarrant et al (1992) used Friesian bulls with average BW of 618 kg (Min, 537 kg; Max 900 kg) at pre-transportation; and in our study, heifers had an average BW of 408 kg (Min, 351 kg; Max 472 kg) at PRET. The lower BW of heifers may cause a decrease in CK units per liter of blood compared to steers with greater BW [24]. In addition, Earley et al (2010) reported an increase in circulating CK levels after 24-hours of transportation in Charolais bulls with BW of 367 ± 35 kg. In that study, before transportation, average CK level was 162.3 U/L, which is lower than in our study at PRET (CTRL, 227.4 U/L; RPM, 266.7 U/L). However, circulating CK after 24-hours transportation was 496 U/L, which is similar to heifers’ POST CK levels measured in this study (CTRL, 406 U/L; RPM, 424.8 U/L) [25]. In conclusion, beyond the rise in glucose and CK levels after transportation, no significant effect was observed due to the addition of RPM in heifers’ diet pre-transportation. Counter to our hypothesis, there was not a greater CK concentration, which could mitigate the effect of muscle fatigue due to greater amounts of creatine phosphate synthetized from RPM administration.

### Gene expression

#### Dystrophin-glycoprotein complex

Dystrophin-glycoprotein complex (DGC) is a key element for the integrity of the sarcolemma, composed primarily of dystrophin and sarcoglycan complexes. The function of DGC is associated with the anchoring of sarcolemma to the actin cytoskeleton [30]. The DGC is formed by the dystroglycan complex, composed by dystrophin, the syntrophins, α- and β-dystroglycan; and sarcoglycan complex (SG), which is formed by four different transmembrane glycoproteins called α-, ß-, γ-, and δ -sarcoglycan [31]. Dystrophin, a cytoskeletal actin-binding protein, is one of the major proteins designed for the integrity of skeletal muscle fibers [32]. The dystroglycan complex is responsible for binding the basal lamina through α-dystroglycan and laminin [33] and attaches dystrophin at its C-terminal domain via β-dystroglycan [31]. At the same time, dystrophin N-terminal binds actin generating a link between the cytoskeleton and the extracellular matrix [34].

Sarcospan (*SSPN*), a transmembrane protein, is a DGC member [35] with a role in stabilizing the sarcolemma. Additionally, *SSPN* may serve as a chaperone in the DGC assembly and may help enhance localization of other adhesion complex proteins [36]. To the best of our knowledge, there is no literature reporting the effects of fatigue on *SSPN* expression. Therefore, it would be possible that CTRL group had more stabilization in the sarcolemma by *SSPN* upregulation between BASE and PRET. However, in a previous study, *SSPN* deficient mice present normal creatine kinase levels, sarcolemma integrity, and force generation aptitude [36].

The gene expression regulation of Syntrophin isoforms is an important factor for the sarcolemma integrity and binding signaling proteins. If one isoform is missed or deleted, another can replace it with no functional effects observed [37]. The absence α-Syntrophin 1 (*SNTA1*) gene in mutant mice did not show reduced force recovery in a fatigue stimulation trial [38]. Mice lacking α- and ß-syntrophins (*SNTA1* and *SNTB1*, respectively) produce a decrease in skeletal muscle function [37]. It is possible to infer that sarcolemma integrity might not be affected by RPM supplementation nor fatigue due to transportation, since both *SNTA1* and *SNTB1* expression did not differ between treatments. Similarly, ß-sarcoglycan (*SGCB*), a gene that encodes for a transmembrane protein in the sarcoglycan complex, showed a similar pattern of expression in comparison to *SNTA1* and *SSPN*, indicating its possible correlation with the whole Dystrophin-Glycoprotein complex. Because *SSPN* was the only gene that showed a treatment effect x time interaction in the Dystrophin-Glycoprotein complex, it would not be accurate to declare that the sarcolemma maintains its stability in CTRL group during transportation due to the unique upregulation of *SSPN* at PRET. Consequently, administration of RPM may not alter skeletal muscle membrane integrity in growing heifers. Further analysis of more genes related to Dystrophin-Glycoprotein complex and their phenotypic effects will help to fully understand the mechanism behind changes on skeletal muscle membrane’s integrity under this stressful condition.

#### Sarcoplasmic reticulum

The regulation of Ca^2+^ ions in muscle cells has been widely studied in different species; however, little is known about the effect of fatigue on beef cattle. In a previous study, a downregulation of *CASQ1* protein expression in mice after an endurance trial [39] was reported. These results are consistent with our study for the CTRL group, because there was a downregulation of *CASQ1* between PRET and POST. Similarly, reduction in activity of *ATP2A1* in fatigued skeletal muscle was reported in rats [40] and humans [41].Even though, we did not measure Ca^2+^ levels in skeletal muscle, the downregulation of *CASQ1*, *ATP2A1*, and *SYPL2* in CTRL during transportation may be related to Ca^2+^ depletion after a repetitive use of muscle [42]. These responses were not observed in heifers that received RPM between BASE and PRET. It has been shown that, after a methylation process of sarcolemma throughout SAM as a methyl donor agent, the Na^+^-Ca^+^ exchange is inhibited in the cardiac muscle. In skeletal muscle, phospholipid methyltransferase activity is highly localized in sarcoplasmic reticulum and present to a lesser extent in sarcolemma. Methylation of phosphatidylethanolamine modulates sarcoplasmic reticulum phospholipid composition, which in turn alters the energetic efficiency of SERCA (Ca^2+^ uptake / ATP hydrolysis) ion pump, decreasing the skeletal muscle metabolic rate [43–45]. Our results can be explained by a possible inhibition of Ca^2+^ uptake under an excess of methylation in the sarcoplasmic reticulum of RPM.

The administration of RPM downregulated only one of the genes that we selected to study the sarcoplasmic reticulum complex, *SYPL2*. It is possible that the methyl groups provided by RPM inhibited *SYPL2* expression. Nagaraj et al (2000) reported that wild-type mice and mice lacking MG29, the protein that *SYPL2* encodes, exhibited lower force generation after fatigue in the diaphragm, a skeletal mixed-fiber muscle like the *Longissimus Dorsi* [46]. In addition, mice lacking *SYPL2* did not show decreased force generation after fatigue compared with wild-type mice, indicating the effect of MG29 can be compensated in a muscle fatigue scenario for that species. In RPM, *ATP2A1* and *CASQ1* remained unchanged among time points, suggesting that the greater availability of methyl groups does not modify Ca^2+^ regulation in skeletal muscle or inhibits the expression of genes related to sarcoplasmic reticulum. Skeletal muscle fatigue impairs Ca^2+^ uptake as previously described [47]. Down-regulation in the expression of all the genes analyzed related to sarcoplasmic reticulum between PRET and POST in CTRL may suggest that Ca^2+^ metabolism could be impaired under fatigue conditions. In contrast, RPM did not show a significant difference between PRET and POST. Thus, the stabilization of the expression of genes related to sarcoplasmic reticulum could potentially explain a better Ca^2+^ uptake by the skeletal muscle [47, 48].

#### Creatine synthesis pathway

To the best of our knowledge, there are no published nutrigenomics studies of muscle fatigue during transportation stress in cattle, and because of this, we utilized information from other speciesCreatine biosynthesis occurs mainly in the kidney, pancreas and liver, and involves two steps [49]. First, glycine amidinotransferase (*GATM* or *AGAT*) catalyzes the transamidination of guanidine group from arginine to glycine, producing ornithine and guanidinoacetate (GAA) [50]. Second, guanidinoacetate methyl transferase (*GAMT*) catalyzes the addition of a methyl group from S-adenosyl methionine (SAM) to GAA, producing creatine and S-adenosyl homocysteine (SAH) [51]. Once creatine is formed, it reaches different tissues via its transporter called solute carrier family 6 member 8 (*SLC6A8*) [52].

In our study, the difference in *GATM* expression can be explained by the maintenance of the expression in RPM at POST, in comparison to CTRL, which was downregulated. In mice, *GATM* upregulation in skeletal muscle does not compensate deficiency in *SLC6A8* [53]. Thus, it is possible that the *GATM* expression in CTRL does not make up for decreased expression of *CKM* and *SLC6A8* at POST.

In addition, *GAMT* deficiency has a negative effect in force maintenance in both, high-intensity electrical stimulation, and lower electrical stimulation frequency in mice [54]. The downregulation of *GAMT* expression in CTRL between PRET and POST would explain a decrease in creatine synthesis due to muscle fatigue since *CKM* expression was downregulated in CTRL for the same period of time.

In skeletal muscle of Thoroughbreds horses, no difference in *CKM* expression was found under moderate and high intensity exercise on a treadmill [55]. Samples were taken before the exercise, immediately after exercise, and 4 hours later. It has been shown that muscle force generation vary depending on the activity of the muscle. For example, muscle generates larger forces at moments of high speeds (i.e. running) compared with lower speed of muscle use (i.e. walking) [56]. Consequently, the velocity of muscle contraction has an important influence in energy use. For example, energy expenditure during an active movement (i.e. walking) is greater compared with less motion activities (i.e. standing) [57]. In our study, animals were not doing an intense exercise but they were certainly contracting theirs muscles in order to maintain equilibrium inside the truck for a long period of time, it is possible to compare these results with ours. However, even though RPM treatment remained stable among time points for *CKM* expression, it is not possible to explain the upregulation in CTRL heifers between BASE and PRET.

Interestingly, *SLC6A8* shows the same pattern as *CKM* expression for both treatments among all time points. Since *SLC6A8* encodes for the major creatine transporter, the decrease of creatine levels produces an inhibition of *SLC6A8* expression, similarly to results reported in previous studies with creatine transporter deficiency in mice [52].

#### Oxidative stress

The generation of free radical elements such as Nitric Oxide (NO^-^) and superoxide (O_2_^-^) is a result of normal cellular metabolism [58]. Superoxide suffers reductions generating reactive oxygen species (ROS) [i.e., O_2_^-^, hydrogen peroxide (H_2_O_2_) and, hydroxyl radicals (OH^-^)] during resting conditions. Nitric oxide can be inactivated by coupling with O_2_^-^, producing the oxidant proxynitrite (ONOO^-^) as a result [59]. Nitric oxide creates reactive nitrogen species (RNS) like peroxynitrous acid (HNO_3_^-^), ONOO^-^ and other nitrogen-derived oxidants [60]. These products are released in low concentration during resting, and they are necessary for normal cellular function [59]. For example, these compounds are involved in muscle regeneration, acting with other elements like growth factors and chemokines. Additionally, ROS promote mitochondriogenesis during exercise, which generates higher ATP levels for cell utilization [61]. However, skeletal muscle generates oxidants at high rates during muscle contraction, and they either became blockers of myogenic differentiation, or cause injuries by oxidative damage [62]. A single bout of exhaustive exercise leads to strong increases in ROS, which cannot be buffered by endogenous antioxidants, particularly in untrained individuals [63]. Therefore, high levels of ROS results in contractile dysfunction and fatigue [64]. In contrast, in our study, a prolonged period of water deprivation and utilization of energy reserves, which occur during long-term transportation of livestock, ultimately result in dehydration and a switch to a heightened gluconeogenic state while sparing muscle protein degradation [65]. The generated state of energy deprivation produces an increase in oxidative stress [66].

Exercise and fatigue increase muscle ONOO^-^ and O_2_^-^ producing an excess of oxidants in the cellular milieu. Nevertheless, cellular defense mechanisms against oxidative damage can effectively degrade these products [67]. Superoxide dismutases (SODs) are oxidoreductases generated by cells as a protection against oxidants. They are capable of spontaneous dismutation of O_2_^-^ into H_2_O_2._ Consequently, they also help reducing the production of ONOO^-^.

Mammals present three isoforms of SOD, and they are differentiated by their composition and site of action. The cytosolic *SOD1* (Cu/ZnSOD) is the main intracellular dismutase, which has enzymatic activity dependent on the presence of copper and zinc [68]. The mitochondrial *SOD2* (MnSOD) has enzymatic activity dependent on the presence of manganese. Manganese plays a catalytic role of the dismutation of O_2_^-^ to H_2_O_2_, similar to *SOD1* [59]. The major site of O_2_^-^ generation is the mitochondrial respiratory chain; consequently, *SOD2* is a very important regulator of ROS equilibrium in the cell [69]. Finally, an extracellular dismutase called *SOD3* (ecSOD) catalyzes ROS in the extracellular matrix. It is mostly expressed in internal organs and blood vessels. Skeletal muscle does not present high expression of *SOD3*, thus, it was not subject to analysis in our study [59, 70].

In our study, expression of *SOD1* and *SOD2* was significantly activated in CTRL heifers during the pre-conditioning period compared to RPM heifers. The lack of response in RPM heifers could be interpreted as a better controlled oxidant—antioxidant balance within skeletal muscle. We base our hypothesis on the observation that regular exercise appears to gradually increase the level of adaptation by the repeated activation of antioxidant genes and proteins [71]. We believe that after a continuous contraction of muscles during the 22 hours on the trailer, RPM heifers responded better than CTRL heifers in the adaption to a stressful situation and, they were able to reach homeostasis in their ROS production by the end of the study. Thus, activation of *SOD1* and *SOD2* was not in demand.

As reported in our study, BW losses of 8% in RPM, and 10% in CTRL were caused by the transportation, and up to 50% of that BW loss may be tissue partitioning [18]. During the 45-day preconditioning period with RPM supplementation, most ROS-related genes had an upregulation pattern in CTRL heifers. Methionine is a precursor of S-adenosylmethionine and hydrogen sulfide, which alleviate oxidant stress and protect the tissue from the damage [72]. Therefore, RPM supplementation might prevent an oxidative stress response in the RPM group before and after transportation contrary to CTRL, which showed an upregulation in ROS-related genes during the same period of time. The upregulation in CTRL compared with RPM before transportation may be related to a lower antioxidant capacity due to a minor methionine residues content in skeletal muscle cells. Methionine residues are known as ROS scavengers [73]. The greater expression of superoxide agents in CTRL as compared to RPM may be related to the oxidative stress imbalance due to tissue partitioning in heifers’ adipose and skeletal muscle tissues, due to the long-duration transportation without access to water and feed [74, 75].Furthermore, the occurrence of oxidative stress due to starvation has been reported in different species [76–78]. Fasting rats had a higher hepatic oxidative stress imbalance due to different processes of tissue degradation such as lipid peroxidation, protein misfolding, and glycolysis [79]. In addition, ROS generation increased in skeletal muscle after 24 hours of fasting in mice [80].

Metabolic stress inherent to endurance exercise has been demonstrated to stimulate mitochondrial biogenesis [81]. Mitochondrial synthesis is stimulated by the *PGC-1α*—*NRF1*—*TFAM* pathway [63]. Therefore, *PGC-1α* is the first stimulator of mitochondrial biogenesis [82], and its expression increases with exercise training [83]. *PGC-1α* is a key player in the adaptation of muscle cells to exercise [63]. Oxidative stress increases expression of *PGC-1α* [84], and *PGC-1α* controls cellular antioxidant homeostasis by stimulating *SOD2*, catalase, glutathione peroxidase 1 (GPx1), and uncoupling protein (*UCP*) [85]. In our study, neither administration of RPM nor transportation stress affected *PGC-1α* expression, although the peak in *PGC-1α* expression at PRET in CTRL heifers leads us to suggest that RPM could be producing an inhibition in the expression of this gene. Furthermore, the presence of additional methionine could be producing a more balanced environment in the RPM heifer’s skeletal muscle, where the activation of the oxidative stress-related gene network was not required. In contrast to our results, *PGC-1α* is upregulated in liver of fasting mice [86]. However, *PGC-1α* stabilization in RPM during transportation could mean that tissue partitioning caused by fasting metabolism was not related to the expression of this gen. Furthermore, the effect of mice lacking *PGC-1α* on substrate utilization from fed to fasted state was not relevant [87]. This could explain the lack of significant differences in *PGC-1α*.

The generation of nitric oxide (NO) in endothelial cells is produced by nitric oxide synthetase Type III or eNOS, which is encoded by *NOS3*. The presence of O_2_^-^ can stimulate the development of fatigue. Thus, the inactivation of O_2_^-^ by NO may be protective for the muscle cell [88]. Our results showed that the addition of RPM did not modify the expression of *NOS3*, suggesting that O_2_^-^ remained unchanged among time points and an upregulation of the gene expression might not have been required. There could be a possible relationship between mithocondrogenesis, for *PGC-1α* and *NOS3*. Nisoli et al (2014) supports our results, showing that eNOS -/- muscle cells have lower mitochondriogenesis activity, and lower expression of *PGC-1α* [89].

Finally, *NQO1* is a member of an antioxidant defense system. An acute bout of exercise at sufficient intensity activates this antioxidant enzyme, among others [90]. In contrast, in patients with chronic fatigue syndrome, *NQO1* was downregulated [91]. Similar to other antioxidant genes assessed in this study, *NQO1* expression remained unchanged among time points in RPM heifers. The higher concentration of available methyl groups provided by the addition of RPM could decrease ROS production. Consequently, the activation of *NQO1* would not be necessary for the muscle cells after the transportation simulation was performed.

#### DNA methylation

DNA methylation is the major regulator of epigenetics in the genome of mammalians by the covalent addition of a methyl group (-CH_3_) to cytosine in combination of CpG dinucleotide called “CpG” island [92]. Enzymes capable of adding methyl groups to the hemimethylated and unmethylated DNA are called DNA methyltransferase (DNMT), like *DNMT1* and *DNMT3A*, respectively [93]. Thus, *DNMT1* and *DNMT3A* gene expression in skeletal muscle would potentially explain epigenetic changes due to muscle fatigue. *DNMT3A* presents a catalytic activity of *de novo* DNA methyltransferases while, *DNMT1* works maintaining methylation of the CpG sites [94]. Furthermore, a cooperation between *DNMT1* and *DNMT3B* was observed in humans; when disrupted, DNA methylation was decrease by 95% in colon rectal cancer cell [95].

The addition of RPM to the diet increases methionine availability [96]. Consequently, it can be transferred to S-adenosylmethionine (SAM), which is a molecule considered as the universal methyl donor [97]. Similar to our results, Osorio et al. (2014) did not obtain a significant difference in hepatic *DNMT1* expression in periparturient dairy cows under Smartamine® supplementation. Furthermore, in contrast to our results, hepatic *DNMT3A* showed a greater expression after 11 days under Smartamine® supplementation during the pre-partum period [98]. However, the difference was mitigated after calving, and no difference was obtained from 11 to 31 days of supplementation. In addition, there was a similar pattern of expression of *DNMT1* and *DNMT3A* in CTRL across time points, similar to results from Rhee et al (2002). Although, this pattern was not observed in RPM [95].

The *AHCY* gene provides instructions for producing the enzyme S-adenosylhomocysteine hydrolase, an important inhibitor of the majority of the methytransferases, affecting DNA methylation, RNA, and protein methylation in humans [99]. Specifically, S-adenosylhomocysteine hydrolase controls the chemical reaction that converts S-adenosylhomocysteine to adenosine and homocysteine [100]. This chemical reaction also plays an important role in regulating the addition of methyl groups to other compounds (i.e., methylation) [101]. Therefore, the principal role of *AHCY* in metabolism is to hydrolyse and efficiently remove S-adenosylhomocysteine, the by-product of transmethylation reactions and one of the most potent methyltransferase inhibitors [102]. S-adenosylhomocysteine hydrolase (*AHCY*) is the only mammalian enzyme able to hydrolyze S-adenosyl-l-homocysteine, and it binds to *DNMT1* during DNA replication [101]. Hypermethylation of the genome can occur due to *DNMT1* overexpression caused by *AHCY* regulation in vitro [101]. Furthermore, in isolated guinea pig heart muscles, adrenaline secretion inhibits S-adenosylhomocysteine hydrolase activity by a calcium mediated mechanism [103].

Stress can also directly influence the transcriptional regulation at the epigenetic level. In a previous study, prenatal transportation stress in Brahman bull calves was assessed at the methylome level. Results showed at least 10% more methylation in stressed calves as compared to controls [104]. In our study, RPM produce the inhibition of *AHCY* during preconditioning. However, under the stress of transportation, *AHCY* had activation in RPM heifers, coinciding with *DNMT1* upregulation. Furthermore, in a previous study, the transcriptional repression of *DNMT1* by glucocorticoid exposure is considered as a proxy for stress response [105]. Hypermethylation plays a role in controlling the physiological response to stressors by regulating the release of glucocorticoids in response to challenges [106]. Our results indicate that preconditioning with RPM supplementation might produce an activation of the process of DNA methylation during transportation stress. The animal’s benefit due to this metabolic response (i.e., hypermethylation) to long-term transportation stress still needs to be elucidated.

## Conclusion

There was not a significant reduction in shrink with RPM; however, the utilization of more animals in further studies, could make this difference significant. In addition, serum glucose, creatine kinase and cortisol levels were not affected by the administration of RPM, suggesting that the catalytic processes generated due to high stress level could not be diminished by a greater methionine availability.

Contrary to our hypothesis, rumen-protected methionine during a preconditioning period of 45 days does not have an effect in *CKM* neither genes related to creatine synthesis in beef cattle transported for a period of 22 hours. Expression of genes related to muscle integrity and fatigue, creatine synthesis, sarcoplasmic reticulum and Dystrophin-glycoprotein complex were not affected by the administration of RPM in beef heifer’s skeletal muscle. However, our results indicate that administration of 8 gr/hd/ day of RPM could promote a better response to transportation stress in heifer’s skeletal muscle due to a possibly more stable redox balance after long-duration transportation. Perhaps, evaluating the *Semitendinosus* muscle, which might suffer more tension during transportation, would make these results more clear. However, we wanted to focus on the ribeye area (i.e., *Longissimus dorsi*) where the most expensive beef cuts are located. In conclusion, preconditioning growing Angus × Simmental heifers with RPM pre-transportation may have an effect on oxidative stress (*SOD2*), DNA methylation (*DNMT3A*) and hypermethylation (*AHCY*).

## Supporting information

S1 Fig Average daily gain of beef heifers that received rumen-protected methionine (RPM) and heifers control (CTRL) that did not received RPM. (DOCX)

S1 Table Overall least mean squares values for expression of genes analyzed in *Longissimus dorsi* muscle of Angus-Simmental heifers from CTRL and RPM. (DOCX)

S2 Table Quantitative real time PCR performance among the 20 genes measured in skeletal muscle samples. (DOCX)

S3 Table Gene ID, GenBank accession number, hybridization position, sequence and amplicon size of primers for *Bos taurus* used to analyze gene expression by RT-qPCR. (DOCX)

S4 Table Sequencing results of PCR products from primers of genes designed for this experiment. Best hits using BLASTN (http://www.ncbi.nlm.nih.gov) are shown.
(DOCX)

S5 Table Sequencing results of genes using BLASTN from NCBI (http://www.ncbi.nlm.nih.gov) against nucleotide collection (nr / nt) with total score. (DOCX)
