# Supplementary material for: Preconditioning beef cattle for long-duration transportation stress with rumen-protected methionine supplementation: A nutrigenetics study
Source: PLoS One. 2020 Jul 2;15(7):e0235481. doi: 10.1371/journal.pone.0235481 (PMC7332072; doi:10.1371/journal.pone.0235481)
Supplement: S1 Fig — (DOCX) [file pone.0235481.s001.docx]

**S1 Figure.** Average daily gain of beef heifers that received rumen-protected methionine (RPM) and heifers control (CTRL) that did not received RPM**.**

**
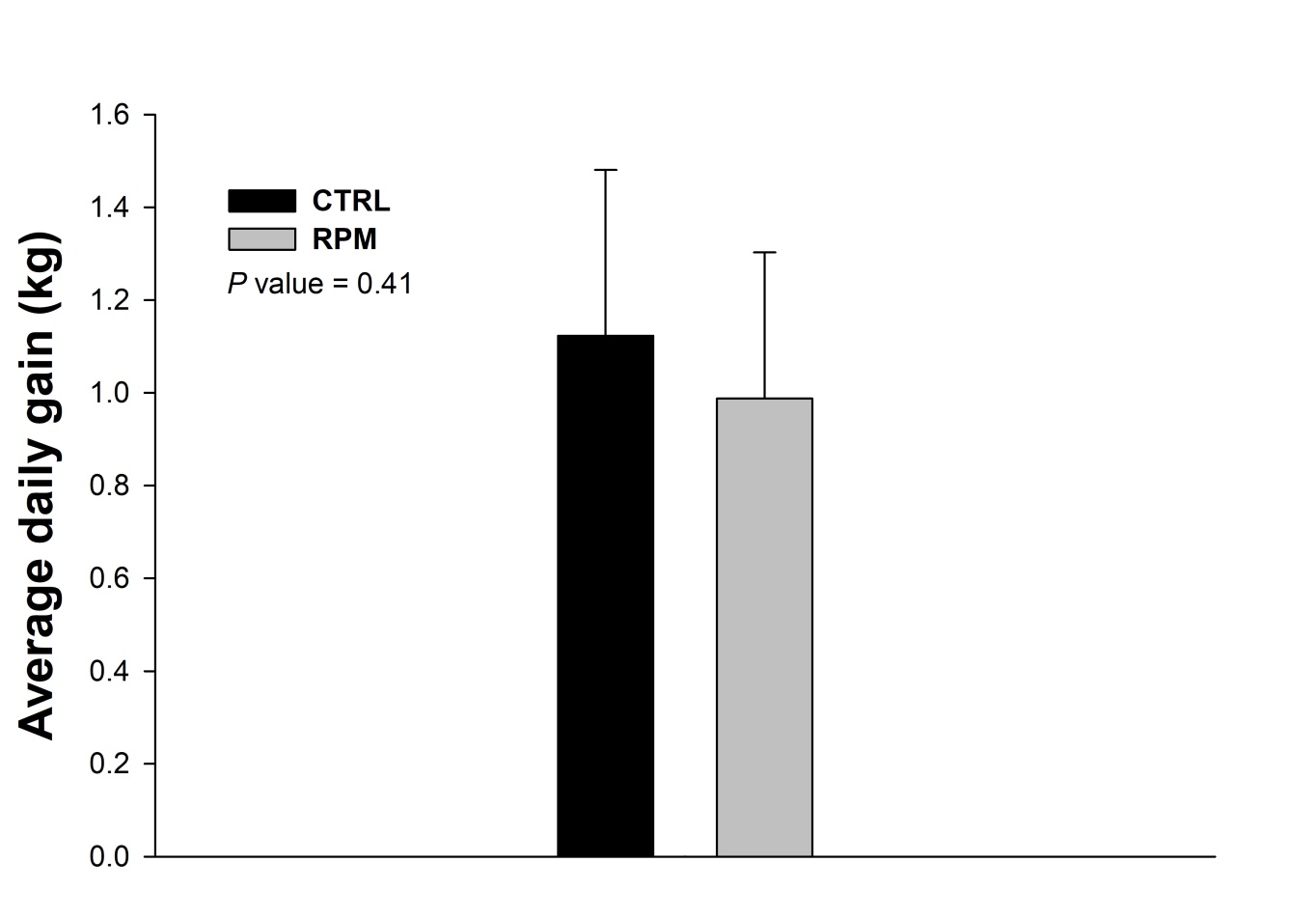
**
