# Supplementary material for: Preconditioning beef cattle for long-duration transportation stress with rumen-protected methionine supplementation: A nutrigenetics study
Source: PLoS One. 2020 Jul 2;15(7):e0235481. doi: 10.1371/journal.pone.0235481 (PMC7332072; doi:10.1371/journal.pone.0235481)
Supplement: S1 Table — (DOCX) [file pone.0235481.s002.docx]

**S1 Table.** Overall least mean squares values for expression of genes analyzed in *Longissimus dorsi* muscle of Angus-Simmental heifers from CTRL and RPM.

|  |  | **PRET** | | **POST** | | **SEM** | ***P -* value** | | |
| --- | --- | --- | --- | --- | --- | --- | --- | --- | --- |
| **Pathway** | **Gene** | **CTRL** | **RPM** | **CTRL** | **RPM** |  | **Trt** | **Time** | **Trt ×Time** |
| Dystrophin-Glycoprotein complex | *SGCB* | 1.64 | 1.03 | 0.97 | 1.11 | 0.14 | 0.694 | 0.017 | 0.001 |
|  | *SNTA1* | 1.19 | 0.77 | 0.67 | 0.74 | 0.09 | 0.273 | 0.001 | 0.001 |
|  | *SNTB1* | 0.93 | 1.25 | 1.23 | 1.62 | 0.33 | 0.361 | 0.025 | 0.337 |
|  | *SSPN* | 1.54 | 0.70 | 0.71 | 0.77 | 0.09 | 0.050 | 0.001 | 0.001 |
| Sarcoplasmic Reticulum | *ATP2A1* | 1.74 | 0.85 | 0.99 | 1.15 | 0.14 | 0.257 | 0.729 | 0.001 |
|  | *CASQ1* | 1.82 | 0.88 | 0.60 | 0.67 | 0.11 | 0.301 | 0.001 | 0.001 |
|  | *SYPL2* | 1.61 | 0.72 | 0.60 | 0.60 | 0.10 | 0.140 | 0.001 | 0.001 |
| Creatine Synthesis Pathway | *GAMT* | 1.02 | 0.84 | 0.44 | 0.58 | 0.09 | 0.891 | 0.001 | 0.026 |
|  | *GATM* | 1.09 | 0.88 | 0.56 | 1.01 | 0.15 | 0.404 | 0.001 | 0.003 |
|  | *AHCY* | 1.04 | 0.65 | 1.25 | 1.05 | 0.08 | 0.038 | 0.001 | 0.001 |
|  | *SLC6A8* | 1.30 | 0.73 | 0.78 | 0.75 | 0.12 | 0.222 | 0.071 | 0.001 |
|  | *CKM* | 1.61 | 0.81 | 0.88 | 0.97 | 0.11 | 0.111 | 0.295 | 0.001 |
| DNA methylation | *DNMT1* | 1.50 | 1.15 | 1.28 | 1.72 | 0.17 | 0.947 | 0.001 | 0.047 |
|  | *DNMT3A* | 1.34 | 0.73 | 0.74 | 0.80 | 0.09 | 0.048 | 0.001 | 0.001 |
| Oxidative Stress | *SOD2* | 1.40 | 0.80 | 1.09 | 0.88 | 0.08 | 0.013 | 0.802 | 0.001 |
|  | *NQO1* | 1.67 | 0.94 | 0.79 | 0.68 | 0.15 | 0.122 | 0.001 | 0.001 |
|  | *SOD1* | 2.59 | 1.19 | 1.95 | 1.25 | 0.32 | 0.835 | 0.001 | 0.039 |
|  | *NOS3* | 2.16 | 1.04 | 4.11 | 2.47 | 0.64 | 0.706 | 0.001 | 0.038 |
|  | *PGC1a* | 3.02 | 0.88 | 1.40 | 1.26 | 0.56 | 0.996 | 0.016 | 0.195 |
|  | *NFKB1* | 3.03 | 1.41 | 3.07 | 1.86 | 0.52 | 0.760 | 0.001 | 0.390 |
