# Supplementary material for: Preconditioning beef cattle for long-duration transportation stress with rumen-protected methionine supplementation: A nutrigenetics study
Source: PLoS One. 2020 Jul 2;15(7):e0235481. doi: 10.1371/journal.pone.0235481 (PMC7332072; doi:10.1371/journal.pone.0235481)
Supplement: S2 Table — (DOCX) [file pone.0235481.s003.docx]

**S2 Table.** Quantitative real time PCR performance among the 20 genes measured in skeletal muscle samples

1- The median is calculated considering all time points and all steers. 2- The median of ∆Ct is calculated as [Ct gene – geometrical mean of Ct internal controls] for each time point and each steer. 3- Slope of the standard curve. 4- R2 stands for the coefficient of determination of the standard curve. 5- Efficiency is calculated as [10(-1 / Slope)]. 6- relative mRNA abundance = 1/ Efficiency Median ∆Ct. 7- 1/E∆Ct = relative mRNA abundance/∑relative mRNA abundance.
