# Supplementary material for: Preconditioning beef cattle for long-duration transportation stress with rumen-protected methionine supplementation: A nutrigenetics study
Source: PLoS One. 2020 Jul 2;15(7):e0235481. doi: 10.1371/journal.pone.0235481 (PMC7332072; doi:10.1371/journal.pone.0235481)
Supplement: S3 Table — (DOCX) [file pone.0235481.s004.docx]

**S3 Table.** Gene ID, GenBank accession number, hybridization position, sequence and amplicon size of primers for *Bos* *taurus* used to analyze gene expression by RT-qPCR.

^1^ Primer direction (F – forward; R – reverse) and hybridization position on the sequence.

^2^ Exon-exon junctions are underlined.

^3^ Amplicon size in base pair (bp).
