# Supplementary material for: Preconditioning beef cattle for long-duration transportation stress with rumen-protected methionine supplementation: A nutrigenetics study
Source: PLoS One. 2020 Jul 2;15(7):e0235481. doi: 10.1371/journal.pone.0235481 (PMC7332072; doi:10.1371/journal.pone.0235481)
Supplement: S4 Table — Best hits using BLASTN (http://www.ncbi.nlm.nih.gov) are shown. (DOCX) [file pone.0235481.s005.docx]

**S4 Table.** Sequencing results of PCR products from primers of genes designed for this experiment. Best hits using BLASTN (http://www.ncbi.nlm.nih.gov) are shown.
