# Supplementary material for: Preconditioning beef cattle for long-duration transportation stress with rumen-protected methionine supplementation: A nutrigenetics study
Source: PLoS One. 2020 Jul 2;15(7):e0235481. doi: 10.1371/journal.pone.0235481 (PMC7332072; doi:10.1371/journal.pone.0235481)
Supplement: S5 Table — (DOCX) [file pone.0235481.s006.docx]

**S5 Table.** Sequencing results of genes using BLASTN from NCBI (http://www.ncbi.nlm.nih.gov) against nucleotide collection (nr / nt) with total score.

| **Gene** | **Best hit in NCBI** |
| --- | --- |
| *AHCY* | [Bos taurus adenosylhomocysteinase (AHCY), mRNA](https://blast.ncbi.nlm.nih.gov/Blast.cgi#alnHdr_77735582) |
| *ATP2A1* | Bos taurus ATPase sarcoplasmic/endoplasmic reticulum Ca2+ transporting 1 (ATP2A1), mRNA |
| *CASQ1* | Bos taurus calsequestrin 1 (CASQ1), mRNA |
| *CKM* | Bos taurus creatine kinase, M-type (CKM), mRNA |
| *DNMT1* | Bos taurus DNA methyltransferase 1 (DNMT1), mRNA |
| *DNMT3A* | Bos taurus DNA methyltransferase 3 alpha (DNMT3A), mRNA |
| *GAMT* | Bos taurus guanidinoacetate N-methyltransferase (GAMT), mRNA |
| *GATM* | [Bos taurus glycine amidinotransferase (GATM), mRNA](https://blast.ncbi.nlm.nih.gov/Blast.cgi#alnHdr_114052740) |
| *MTG1* | Bos taurus mitochondrial ribosome associated GTPase 1 (MTG1), mRNA |
| *NFKB1* | [Bos taurus nuclear factor kappa B subunit 1 (NFKB1), mRNA](https://blast.ncbi.nlm.nih.gov/Blast.cgi#alnHdr_115497301) |
| *NOS3* | [Bos taurus nitric oxide synthase 3 (endothelial cell) (NOS3), mRNA](https://blast.ncbi.nlm.nih.gov/Blast.cgi#alnHdr_317008622) |
| *NQO1* | Bos taurus NAD(P)H quinone dehydrogenase 1 (NQO1), mRNA |
| *PGC1α* | PREDICTED: Bos taurus PPARG coactivator 1 alpha (PPARGC1A), transcript variant X1, mRNA |
| *RPS15A* | Bos taurus ribosomal protein S15a (RPS15A), mRNA |
| *SGCB* | Bos taurus sarcoglycan beta (SGCB), mRNA |
| *SLC6A8* | Bos taurus solute carrier family 6 member 8 (SLC6A8), mRNA |
| *SNTA1* | Bos taurus syntrophin alpha 1 (SNTA1), Mrna |
| *SNTB1* | P[REDICTED: Bos taurus syntrophin beta 1 (SNTB1), mRNA](https://blast.ncbi.nlm.nih.gov/Blast.cgi#alnHdr_1387208155) |
| *SOD1* | Bos taurus superoxide dismutase 1 (SOD1), mRNA |
| *SOD2* | Bos taurus superoxide dismutase 2 (SOD2), mRNA |
| *SSPN* | Bos taurus sarcospan (SSPN), mRNA |
| *SYPL2* | Bos taurus synaptophysin like 2 (SYPL2), mRNA |
| *UXT* | Bos taurus ubiquitously expressed prefoldin like chaperone (UXT), mRNA |
